# How does community health feature in Global Financing Facility planning documents to support reproductive, maternal, newborn, child and adolescent health and nutrition (RMNAH-N)? insights from six francophone West African countries

**DOI:** 10.1080/16549716.2024.2407680

**Published:** 2024-10-02

**Authors:** Joël Arthur Kiendrébéogo, Orokia Sory, Issa Kaboré, Yamba Kafando, Rosie Steege, Asha S. George, Meghan Bruce Kumar

**Affiliations:** a Department of Public Health, University Joseph Ki-Zerbo, Ouagadougou, Burkina Faso; b Research, Expertise and Capacity Building, Recherche pour la santé et le développement (RESADE), Ouagadougou, Burkina Faso; c Heidelberg Institute of Global Health, Medical Faculty and University Hospital, Heidelberg University, Heidelberg, Germany; d Department of Public Health, Institute of Tropical Medicine, Antwerp, Belgium; e Department of International Public Health institution, Liverpool School of Tropical Medicine, Liverpool, UK; f School of Public Health, University of the Western Cape, Cape Town, South Africa; g Nursing, Midwifery and Health, Northumbria University, Newcastle upon Tyne, UK; h Health Systems and Research Ethics, KEMRI-Wellcome Trust Programme, Nairobi, Kenya

**Keywords:** Global Financing Facility for Women, Children and Adolescents: Examining National Priorities, Processes and Investment, Global Financing Facility, investment case and project appraisal document, community health, RMNCAH-N, francophone West Africa

## Abstract

**Background:**

Community health is key for improving Reproductive, Maternal, Newborn, Child, and Adolescent Health and Nutrition (RMNCAH-N). However, how community health supports integrated RMNCAH-N service delivery in francophone West Africa is under-researched.

**Objective:**

We examined how six francophone West African countries (Burkina Faso, Côte d’Ivoire, Guinea, Mali, Niger, and Senegal) support community health through the Global Financing Facility for Women, Children and Adolescents (GFF).

**Methods:**

We conducted a content analysis on Investment Cases and Project Appraisal Documents from selected countries, and set out the scope of the analysis and the key search terms. We applied an iterative hybrid inductive-deductive approach to identify themes for data coding and extraction. The extracted data were compared within and across countries and further grouped into meaningful categories.

**Results:**

In country documents, there is a commitment to community health, with significant attention paid to various cadres of community health workers (CHWs) who undertake a range of preventive, promotive and curative roles across RMNCAH-N spectrum. While CHWs renumeration is mentioned, it varies considerably. Most community health indicators focus on CHWs’ deliverables, with few related to governance and civil registration. Challenges in implementing community health include poor leadership and governance and resource shortages resulting in low CHWs performance and service utilization. While some countries invest significantly in training CHWs, structural reforms and broader community engagement are lacking.

**Conclusions:**

There is an opportunity to better prioritize and streamline community health interventions, including integrating them into health system planning and budgeting, to fully harness their potential to improve RMNCAH-N.

## PAPER CONTEXT

- **Main findings:** Although community health is a key component of the Investment Cases and the Project Appraisal Documents of most of the six francophone West African countries studied, the level of investment varies considerably between countries, and mostly skewed to community health workers, with very little left over for broader community engagement and oversight processes.
- **Added knowledge:** The study describes community health actors, community health interventions and monitoring within a global health initiative, how they fit into the wider health system, the challenges and weaknesses they face and the measures taken to mitigate them, and how they are budgeted.
- **Global health impact for policy and action:** There is a need to adopt a holistic community health systems approach, rather than one focused mainly on CHWs, to fully harness community health’s potential to improve reproductive, maternal, newborn, child, and adolescent health and nutrition.

## Background

Community health is a cornerstone of primary health care (PHC) and is essential to achieving universal health coverage (UHC) and the Sustainable Development Goals (SDGs) [1,2], especially given the glaring inequalities in the world. From the declaration of Alma Ata in 1978 to the present day, the concept of community health has been widely studied, approached, and defined in different ways [3]. Schneider and Lehmann advocate for the term ‘community health system’ as an integrated but specific component of the health system. They define community health systems as ‘the set of local actors, relationships, and processes engaged in producing, advocating for, and supporting health in communities and households outside of, but existing in relationship to, formal health structures’ [4]. While often defined geographically or by identity, communities can be key drivers of change [5]. As a result, the principle of putting individuals and communities at the centre of identifying their own health problems and needs and designing, planning, and implementing solutions to address them with them is often associated with terms such as ‘community participation’, ‘community engagement’, ‘community mobilization’ or ‘community empowerment’ [6].

With regard to specific programs, community-based healthcare provision is an effective strategy for improving Reproductive, Maternal, Newborn, Child, and Adolescent Health and Nutrition (RMNCAH-N) [7–12]. Over the past two decades, several global initiatives have been launched to support progress in RMNCAH-N. The most recent is the Global Financing Facility for Women, Children and Adolescents (GFF) Trust Fund, launched in July 2015. This fund is intended to be catalytic, results-oriented and country-led, bringing together diverse sources of domestic and external financing to support national RMNCAH-N priorities. In particular, the GFF Trust Fund is operationally linked to World Bank (WB) funding through the International Development Association (IDA) or the International Bank for Reconstruction and Development (IBRD) [13]. To identify national RMNCAH-N priorities and guide their investments under the GFF, countries first develop an Investment Case (IC). To promote broad national ownership and equity, the GFF proposes that the development of the IC be inclusive, multi-sectoral, and evidence-based to ensure that targets (e.g. newborns and adolescents) or areas (e.g. postpartum family planning, nutrition, gender-based violence or civil registration and vital statistics) that are typically underfunded or neglected are adequately included [14,15]. Project Appraisal Documents (PADs) are the other key planning document of the GFF. They ideally reflect a subset of the national priorities outlined in the IC and provide financial details for GFF grants in partnership with WB investments through IDA or IBRD funds. As GFF planning documents, the IC and PAD should include evidence-informed strategies or approaches for improving RMNCAH-N, such as community-based healthcare.

This paper is part of research conducted by the Countdown to 2030 health Policy and Systems Group, which previously conducted an independent content analysis of GFF policy documents related to adolescent health [16]. In response to the need for further analysis of GFF processes and attention to community health, as authors based primarily in francophone West Africa, we sought to understand how the six francophone West African countries included in the GFF at the time of writing (Burkina Faso, Côte d’Ivoire, Guinea, Mali, Niger, and Senegal) addressed community health in GFF planning documents and the possible implications of these investments on strengthening community health systems in the region, particularly given the mixed results of the Bamako Initiative [17,18].

While some of these countries have strong community health systems, the scientific literature on community health with an integrated RMNCAH-N perspective in the West African francophone region remains scarce and fragmented, providing opportunities for further improvement and investment. The current literature focuses on specific interventions delivered at the community level, such as family planning [19–21] or disease management, including HIV [22,23], neglected tropical diseases [24,25], malaria [26–29], and health emergencies such as Ebola [30]. Yet, it fails to take a holistic community health systems approach, as defined above [4].

We aimed to generate evidence to inform community health approaches towards RMNCAH-N interventions in our region. This will support francophone West African countries on their path to UHC by providing evidence to support strengthening investment in community health through funding mechanisms such as the GFF.

## Methods

Our study was descriptive and exploratory. We conducted a content analysis of GFF planning documents (ICs and PADs) for the six francophone West African countries included in the GFF by October 2023. All these countries have predominantly agricultural economies, with Côte d’Ivoire’s being the strongest and most diversified with a gross domestic product (GDP) of USD 70.02 billion in 2022 [31]. Senegal, with a GDP of USD 27.68 billion in 2022 [31], also has a diversified economy with a dynamic services sector and oil projects under development. Guinea has vast mineral resources, but poor infrastructure and governance limit its economic potential; its GDP was USD 21 billion in 2022 [31]. Burkina Faso, Mali and Niger are facing major security problems, particularly jihadist attacks, which are hampering their economic development. Their GDP in 2022 were USD 18.82 billion, USD 18.83 billion, and USD 15.34 billion, respectively, [31].

Table 1 provides the core RMNCAH-N impact indicators and the core health financing indicators defined by the GFF, for the six countries included in this study.

**Table 1.** GFF core RMNCAH-N impact and health financing indicators.

	Burkina Faso	Côte d’Ivoire	Guinea	Mali	Niger	Senegal
GFF core RMNCAH-N impact indicators						
Maternal Mortality Ratio (per 100,000 live births)	198.0 (2021)	385.0 (2021)	550.0 (2016)	325.0 (2018)	520.0 (2015)	236.0 (2017)
Under 5 Mortality Rate (per 1,000 live births)	48.0 (2021)	74.0 (2021)	111.0 (2018)	101.0 (2018)	123.0 (2021)	37.0 (2019)
Neonatal Mortality Rate (per 1,000 live births)	18.0 (2021)	30.0 (2021)	32.0 (2018)	33.0 (2018)	43.0 (2021)	21.0 (2019)
Stillbirths Rate (per 1,000 total births)	–	–	13.4 (2018)	12.0 (2018)	16.6 (2012)	19.8 (2019)
Adolescent Birth Rate − 15–19 (per 1,000 women)	93.0 (2021)	96.0 (2021)	128.0 (2021)	145.0 (2021)	133.0 (2021)	71.0 (2019)
Percent of births <24 months after the preceding birth (%)	16.1 (2018)	14.9 (2011)	16.4 (2018)	22.8 (2018)	24.3 (2018)	14.2 (2018)
Stunting among children under 5 years of age (%)	22.6 (2021)	23.4 (2021)	25.6 (2022)	26.9 (2018)	47.0 (2021)	17.9 (2019)
Moderate and severe wasting among children under 5 years of age (%)	10.6 (2021)	8.4 (2021)	6.7 (2022)	8.8 (2018)	33.7 (2021)	8.1 (2019)
GFF core health financing indicators						
Share of government budget allocated to health (%)	13.5 (2021)	5.0 (2022)	7.6 (2022)	5.8 (2022)	4.4 (2021)	5.7 (2020)
Health budget execution (%)	99.0 (2021)	95.3 (2022)	59.0 (2022)	95.3 (2022)	67.9 (2021)	95.0 (2020)
Share of health expenditure going to frontline providers (%)	–	–	26.1 (2020)	15.4 (2022)	12.7 (2021)	–
Domestic General Government Health Expenditure (DGGHE), per capita (US$)	23.5 (2020)	31.3 (2020)	11.2 (2020)	12.2 (2020)	14.7 (2021)	14.8 (2019)
Sum of Domestic General Government Health Expenditure (DGGHE) as share of General Government Expenditure (%)	11.5 (2020)	6.7 (2020)	6.1 (2020)	5.7 (2020)	8.0 (2021)	4.3 (2019)
Sum of Out-of-pocket spending on health, per capita (US$)	18.8 (2020)	27.2 (2020)	22.2 (2020)	10.2 (2020)	15.2 (2020)	30.1 (2019)

Source: The GFF country Data Portal.

We adapted the READ approach, proposed by Dalglish *et al*. [32] for qualitative policy analysis in the following steps:

### Readying our materials (READ)

We downloaded the ICs and PADs available by October 2023 for each included country from the GFF and WB websites. The titles and years of publication including the periods covered by the documents are presented in Table 2. The research team worked together to develop a common understanding of the scope of the analysis and agreed on the key search terms and frameworks to be used in analysing these documents.

**Table 2.** Overview of the ICs and PADs examined.

Countries	ICs		PADs	
	Title	Year of publication (period covered)	Title	Year of publication (period covered)
Burkina Faso	Improving reproductive, maternal, newborn, child and adolescent health, nutrition and civil registration and vital statistics	June 2019, revised in June 2020 Period covered not specified	Health Services Reinforcement Project	June 2018 (2018–2023) Period covered extended to 2024
Côte d’Ivoire	Health financing – Investment case	April 2019 (2020–2023)	Strategic Purchasing and Alignment of Resources & Knowledge in Health Project (SPARK–health)	March 2019 (2019–2025)
Guinea	Investment case for reproductive, maternal, neonatal, child and adolescent health (RMNCAH) in Guinea	Year of publication not specified (2017–2020)	Guinea Health Service and Capacity Strengthening Project	April 2018 (2018–2023)
Mali	Investment case for reproductive, maternal, newborn, adolescent health and nutrition	Year of publication not specified (2019–2023)	Mali – Accelerating Progress Towards Universal Health Coverage	February 2019 (2019–2023)
Niger	Niger’s investment case for reproductive, maternal, neonatal, child and adolescent health and nutrition	March 2022 (2022–2026)	Niger, Improving Women’s and Girls’ Access to Improved Health and Nutrition Services in the Priority Areas Project – LAFIA–IYALI (Phase 1)	August 2021 (2021–2026) Expected Project Closing Date = June 2026 Expected Program Closing Date = June 2036
Senegal	Reducing maternal, neonatal, infant and child, adolescent and youth mortality	June 2019 Period covered not specified	Investing in Maternal, Child and Adolescent Health	September 2019 (2019-2024)

### Extracting data (READ)

Data were extracted by searching for the following words in French: ‘*communauté*’ (community), ‘*communautaire*’ (community)’, ‘*groupe*’ (group), ‘*association*’ (association), ‘*organisation*’ (organization), and ‘*société civile*’ (civil society). This allowed us to identify compound terms such as ‘community health’, ‘community health worker’, ‘community-based association’, ‘community-based organization’.

Any relevant excerpts relating to these terms were copied into a Word file and then referred to for more in-depth content analysis. From these excerpts, an initial list of research themes was identified inductively. This list was then applied deductively on the ICs and PADs for a more extensive data extraction. This hybrid inductive-deductive approach was used iteratively, and 12 final themes were retained for data coding, as shown in (Box 1).

**Box 1: Themes used for data coding**

1. Definitions and aims of community health

2. Integrating community health into the health system

3. Development of community health in insecure areas

4. Actors involved in the implementation of community health

5. Activities of community actors, e.g. the range of services they provide

6. Role of community health actors in implementing RMNCAH-N related policies/strategies

7. Role of community actors in the Civil registration and vital statistics (CRVS)

8. Incentives for community actors to implement activities

9. Indicators for monitoring the performance of those involved in implementing community health

10. Weaknesses in community health implementation

11. Actions taken to improve or strengthen community health

12. Budget/resources allocated to community health

**Table:** 

Box 1. Themes used for data coding
1. Definitions and aims of community health2. Integrating community health into the health system3. Development of community health in insecure areas4. Actors involved in the implementation of community health5. Activities of community actors, e.g. the range of services they provide6. Role of community health actors in implementing RMNCAH-N related policies/strategies7. Role of community actors in the Civil registration and vital statistics (CRVS)8. Incentives for community actors to implement activities9. Indicators for monitoring the performance of those involved in implementing community health10. Weaknesses in community health implementation11. Actions taken to improve or strengthen community health12. Budget/resources allocated to community health

To ensure the consistency and comparability of the extracted data, two authors (IK and YK) retrieved the data according to the search terms. Where the data conflicted, discussions were held with a third party (JAK) to reach agreement. The extracted data were summarised in a table for each type of document (IC and PAD) across all six countries to facilitate analysis.

### Analysing data and distilling findings (READ)

We analysed the content of the ICs and PADs on community health according to the themes identified and compared them, highlighting the similarities and differences within and across countries. Following this initial analysis, the 12 themes were further grouped into five main categories, which form the component parts of our ‘Results’ section. These include: i) the definition of community health, its objectives and place in the health system; ii) the role of community health actors and their activities; iii) their payments, incentives, and performance monitoring; iv) the main challenges and weaknesses in implementing community health activities and actions for improvement; v) the budget earmarked for funding community health.

### Ethics consideration

No ethical or special permissions were required for this study as it did not involve research on human subjects, and the source documents are publicly available. The authors of this paper are health systems researchers with expertise in community health, but except for Burkina Faso, where not involved in developing the country documents assessed. In Burkina Faso, the first author (JAK) helped develop and write the PAD and played an active role in the initial stages of drafting the IC. However, for the sake of objectivity, the data was extracted by other members of the research team who were not involved in drawing up the documents.

## Results

### Definition and aim of community health and where it fits into the health system

With the exception of Burkina Faso’s IC, no other included document explicitly defined community health. Community health was defined in Burkina Faso’s IC as ‘*health by and for the people, supported by the availability in the community of services provided by selected members of the community*’. Despite the absence of definitions, the ICs and/or PADs in all countries but Senegal indicated that community health is an integral part of the health system and a foundation for primary health care services. Consistent with this, national community health policy or planning documents had been developed (Burkina Faso and Niger) or were under development (Cote d’Ivoire, Mali, and Guinea) in these countries. In terms of operationalizing such commitments, while community health objectives were not mentioned in any of the countries’ ICs, they were mentioned in three of the six PADS reviewed (Côte d’Ivoire, Guinea and Niger) and consisted of bringing health services closer to the community, stimulating demand for, and providing effective community-based services.

In three of the six countries (Burkina Faso, Mali and Niger), the security situation characterized by terrorist attacks was a major concern. This security challenge can hamper health service delivery, and the GFF planning documents of these three countries took this into account. The community approach was identified as a way of reaching as many people as possible and, in particular, bringing high-impact package of community-based interventions to people in remote and insecure areas.

### Community health actors: role and activities

Although there are similarities in content, different names were used in different countries to describe the actors implementing community-based health activities (Box 2). While some define community health workers (CHWs) as those working at community level without formal professionalization, given the broad range of actors mentioned we refer generically to those involved in implementing these activities. No included document gives details of the recruitment criteria for CHWs. Regarding gender, Niger’s PAD describes plans to promote female CHWs to improve women’s representation. In Senegal, mobile midwives are all female.

**Box 2: Overview of the terms used to refer to the actors implementing community health in different countries in the GFF planning documents**

1. Burkina Faso: community-based health workers; community-based organisations; civil society actors

2. Côte d’Ivoire: community health workers

3. Guinée: volunteer community health workers; matrons; community relays; health, sanitation, and hygiene committees; water, sanitation, and hygiene agents

4. Mali: community health workers; community relays; civil society actors, village registration officers

5. Niger: multi-skilled community relays; community workers, community health workers, community relays

6. Senegal: community health actors; community-based organisations; community watch and alert committees; mobile midwives

**Table:** 

Box 2 Overview of the terms used to refer to the actors implementing community health in different countries in the GFF planning documents
1. Burkina Faso: community-based health workers; community-based organisations; civil society actors2. Côte d’Ivoire: community health workers3. Guinée: volunteer community health workers; matrons; community relays; health, sanitation, and hygiene committees; water, sanitation, and hygiene agents4. Mali: community health workers; community relays; civil society actors, village registration officers5. Niger: multi-skilled community relays; community workers, community health workers, community relays6. Senegal: community health actors; community-based organisations; community watch and alert committees; mobile midwives

All country documents, except Côte d’Ivoire IC, detailed activities by CHWs that were partly based on the Integrated Management of Childhood Illness (IMCI) at community level. These activities can be categorized as promotional, preventive and curative. Table 3 shows the activities of CHWs according to this categorization and their targets. CHWs are involved in activities across the RMNCAH-N spectrum, except that activities for adolescents were rarely mentioned. However, the Niger PAD foresees CHWs providing a continuum of RMNCAH-N services with a focus on adolescent girls, through the ‘life-course approach’.

**Table 3.** Overview of CHWs activities, by types of services, in the countries’ ICs and PADs.

	Target			
Type of health service	Reproductive	Maternal	Newborn/Child	Family/Community
Promotional	-Awareness raising on adverse consequences of female genital mutilations (FGM) (PAD of Guinea)	-Home visits to pregnant or post-partum women to refer them to health facilities for antenatal care, delivery, child immunization, postnatal follow-up, and FP (IC of Senegal) -Promotion of maternal nutrition (IC of Burkina Faso)	-Promotion of breast feeding (PAD of Côte d’Ivoire and IC of Burkina Faso)	-Awareness raising on WASH (IC of Guinea) -Identify and report cases of gender-based violence (PADs of Mali and Guinea) -Social mobilization (PADs of Burkina Faso and Guinea) -Promotion of food hygiene (IC of Burkina Faso) -Promotion of hand washing (IC of Mali)
Preventive	-Contraceptive distribution (ICs of Burkina Faso and Niger)	-Distribution of iron + folic acid (IC of Burkina Faso)	-Promotion of vaccinations (PAD of Côte d’Ivoire) -Distribution of anti-malarial drugs (IC of Burkina Faso) -Vitamin A, iodine, and other micronutrients supplementation (PAD of Mali and (IC of Burkina Faso) -Monitoring and detection of malnutrition cases (PADs of Côte d’Ivoire and Mali and IC of Guinea) -Integrated disease surveillance and response, including epidemiological surveillance of diseases covered by the expanded programme on immunization (PAD of Mali)	-Distribution of long-lasting insecticidal nets and deworming medicines (PAD of Côte d’Ivoire and ICs of Burkina Faso and Mali) -Epidemiological surveillance of diseases with epidemic potential, neglected tropical diseases, and unusual events (ICs of Niger and Senegal)
Curative	-Follow-up of women with fistula (IC of Senegal) -Referral of victims of female genital mutilation to health services (PAD of Mali)	-Adapted postnatal care for mothers and newborns (PAD of Mali)	-Treatment of common diseases such as malaria, pneumonia, and diarrhoea, and referral of severe cases to health facilities (PAD of Mali and ICs of Burkina Faso, Guinea, Mali, Niger, and Senegal) -Treatment and monitoring of malnutrition cases (PADs of Côte d’Ivoire and Mali and IC of Burkina Faso)	-Treatment of common diseases and referral of severe cases to health facilities (IC of Guinea) -Monitoring of patients with HIV and tuberculosis (IC of Niger)

In addition to activities specific to the RMNCAH-N continuum, CHWs also were tasked with broader health system responsibilities that impact RMNCAH-N. Indeed, CHWs’ activities included monitoring health care services as well as collecting complaints and evidence of health rights violations in their communities and reporting them to local authorities (as in the Senegal PAD), correctly and regularly completing data management tools and submitting them to health facilities (as in the Mali PAD), and also contributing to civil registration and vital statistics (CRVS) activities (as in the Guinea, Mali, and Senegal ICs, and the Burkina Faso PAD). The latter involved raising household awareness of the importance of birth certificates and ensuring birth registration in villages in Mali, following up on the registration of newborns at the civil registry in Senegal, and supporting the CRVS in general (without further details) in Guinea and Burkina Faso.

### Community health actors: payments, incentives, and activity monitoring

Financial payments or incentives were more often mentioned in PADs than in ICs, and in PADs usually through performance-based financing (PBF) schemes, as was the case in Burkina Faso, Côte d’Ivoire, Guinea, and Mali. In Burkina Faso, a strategic purchasing approach was proposed in addition to PBF, both including mechanisms that link CHWs’ individual payments to the achievement of pre-defined service delivery targets or indicators. The Guinean PAD only mentioned an amount budgeted for the annual salary of CHWs. The Niger PAD did not provide information on financial incentives, but its IC mentioned that CHWs receive a monthly financial allowance of XOF 20,000 (about US$35), of which XOF 15,000 (about US$26) is provided by donors and XOF 5,000 by the government. To date, the government notes no plans to transition these costs to its own budget. Burkina Faso’s IC also mentioned that CHWs receive a financial allowance of XOF 20,000 (about US$35), without specifying where the funding comes from. One form of non-financial incentive was found in Mali’s IC – the organization of community competitions called ‘Healthy Village’ and ‘Healthy School or Institution’, which aim to create fair competition between communities and schools in rural and urban areas to promote sanitation. In Senegal, neither the IC nor the PAD provided information on payments or incentives for CHWs.

Senegal’s IC and PAD did not include any indicators to monitor the performance of CHWs and community health activities nor did the ICs of Côte d’Ivoire and Niger. Almost of all the indicators linked to community service delivery in the other country documents are delivered by CHWs, while the governance and CRVS indicators measure processes or outputs at the community level. The IC and PAD indicators for Burkina Faso and Guinea were completely different, while Mali’s IC indicator also appears in its PAD. Whether these indicators are part of the health management information system or a separate community health information system was not specified. Table 4 provides an overview of community health indicators in the ICs and PADs, presented by thematic category.

**Table 4.** Community health indicators in the ICs and PADs, by thematic category.

Thematic categories		IC	PAD
Provision of services	Child Health	-Percentage of children under five with confirmed malaria treated with ACTs by CHWs (Mali)	-Percentage of children aged 6-59 months screened by community health workers for acute malnutrition (Mali) -Number of children who have received curative services by CHWs for children who suffer from pneumonia, uncomplicated malaria, and diarrhoea (Niger) -Percentage of children under two receiving essential community nutrition in targeted areas (Niger)
	Home visits		-Number of households receiving proactive community case management visits (Mali)
	Family/reproductive health		
	WASH initiatives		
	General services	-Percentage of villages benefiting from a full package of community-based care (TB, HIV, malaria) provided by CHWs (Burkina Faso) -Percentage of villages and districts with CHWs coverage by service delivery norms (Burkina Faso)	-Percentage of nutrition sensitive tested community-based initiatives related to WASH to reduce vulnerability to extremes temperature and water-borne diseases (Niger)
Capacity building			-Number of CHWs receiving training on community-level IYCF and IMCI (Burkina Faso) -Number of community health workers recruited and trained in RMNCH competencies (Guinea) -Percentage of CHWs trained on the complete package (curative, preventive and promotional) vis-à-vis the expected number (Niger)
Governance/accountability			-Citizens and/or communities involved in planning, implementation, evaluation of development programs (Yes/No) (Côte d’Ivoire) -Percentage of beneficiaries feedback through community surveys integrated to inform changes in the PBF design (Niger)
CRVS		-30% of deaths in communities were registered and their causes determined in real time (Guinea) -50% of marriages celebrated in communities were registered at the civil registry office (Guinea)	

### Challenges and weaknesses in implementing community health activities and actions for improvement

All documents mentioned challenges and weaknesses in the implementation of community health services, except for four (IC and PAD of Côte d’Ivoire, IC of Guinea, and PAD of Senegal). Table 5 gives an overview of the main challenges and weaknesses mentioned in the documents. Key challenges ranged across health system dimensions of community health: overall poor leadership and governance, shortage of human resources and other inputs, poor performance of CHWs, and low use of community health services.

**Table 5.** Main challenges and weaknesses in community health reported in countries’ ICs and PADs.

	Burkina Faso	Côte d’Ivoire	Guinea	Mali	Niger	Senegal
IC	-Insufficient coordination, management, planning, monitoring and evaluation of community-based care -Poor quality of community care due to low CHW skills, poor supervision, frequent stock-outs of supplies and drugs -Insufficient collaboration between CHWs and health workers in health facilities	-Not mentioned	-Not mentioned	-Shortage of CHWs, both in quantity and quality -Low availability of inputs and low input supply rate at community level	-Low community interventions coverage due to socio-cultural unacceptability (social norms, beliefs, traditional practices), limited input availability, and insufficient human resources -Poor incentives for CHWs to carry out activities, including in hard-to-reach areas	-Insufficient qualified CHWs -Non-harmonisation of the activities of the community watch and alert committees, which hampers their effectiveness and scale-up
PAD	-Poor community engagement and inadequate community-level surveillance and response structures for infectious diseases and epidemics	-Not mentioned	-Lack of contraceptive products for community dispensing -Health management information system not operational at community level	-Low utilization of community health services -Poor skills/performance of CHWs due to poor training and supervision, lack of basic equipment, and weak financial incentives	-National low coverage by CHWs (42%) -Fragmentation and lack of a comprehensive, integrated and harmonised package of interventions	-Not mentioned

The ICs and PADs of all countries identified several actions that could mitigate the challenges and weaknesses identified and improve community health. This included actions to strengthen and expand the capacity and reach of CHWs, which all countries included in one or both documents. These involved activities directly related to RMNCAH-N and others that support RMNCAH-N. The former mainly included training CHWs to recognize danger signs for mothers and newborns or to deliver the IMCI package in the community, essential family practices, and integrated home-based care in hard-to-reach areas. The latter, which accounted for the majority of activities, mainly included: i) training CHWs in social and behavioral communication, treatment of waterborne diseases, hygiene, and sanitation; ii) motivating CHWs to carry out specific health promotion activities, such as hand washing, distribution and promotion of Long Lasting Insecticidal Nets, and organization of community dialogue sessions; iii) strengthening the capacity of CHWs to use digital tools to manage risks associated with potential climatic events and natural disasters (e.g. increased risk of diarrhoeal diseases in case of flooding).

Other planned actions targeted towards improving community health include: i) building and equipping community health posts to enhance infrastructure availability and establishing community health clubs to carry out household health promotion activities (Burkina Faso IC); ii) strengthening supply chain logistics to improve availability of inputs and medicines (Niger IC and PAD); iii) better engaging communities, local leaders and authorities in supporting health services by involving them in monitoring progress and holding health providers accountable for the quality and responsiveness of services to community needs (Côte d’Ivoire and Guinea ICs).

### Community health budget

Community health was budgeted for in the ICs of all countries except Guinea. Although there was variation between countries in terms of the total cost of funding in ICs for community health, Niger and Burkina Faso had the highest proportions of the total budget allocated to community health, at 23% and 21%, respectively. In Niger, two community health strategies were budgeted: i) strengthening the quantity and quality of human resources and community relays and their equitable deployment in rural and hard-to-reach areas (22% of the IC budget); ii) strengthening community participation in RMNCAH-N services (<1% of the IC budget). In Burkina Faso, the budget covered community health and transversal interventions targeting many other activities, although the latter were not specified in a detailed manner. As for Mali, Côte d’Ivoire and Senegal, 7%, 2% and <1%, respectively of their total IC budget was allocated to community health under the headings of implementation of RMNCAH-N packages, community mobilization and strengthening of community strategies.

Related to PADs, community health was budgeted for in all countries except for Burkina Faso and Côte d’Ivoire. Of those financing community health, in absolute terms Niger allocated the most funding for community activities, US$30 million, and Guinea allocated the least, US$5 million. In these two countries, the money was to be used to support the recruitment, training, supervision, and monitoring/coaching of CHWs. In Niger, it was also planned to provide them with equipment and to monitor and strengthen referral mechanisms between the community and health facilities. In Senegal, the total funding of US$15 million was specifically targeted at community nutrition activities. In Mali, the total amount was US$13 million and aimed at strengthening community health activities and the performance of CHWs.

## Discussion

This study examined how community health was prioritised in strengthening RMNCAH-N in six francophone West African countries through a content analysis of their GFF planning documents. We found that while no country documents used definitions, there was an overall commitment to community health, particularly in crisis settings, even if it was not necessarily articulated through community health objectives. Significant attention is paid to a range of CHWs who undertake varied preventive, promotive and curative roles across RMNCAH-N spectrum, highlighting the centrality of their role in community health. While renumeration is mentioned in various country documents, it varies considerably. Many of the community health indicators used are related to CHWs deliverables, except for a few related to governance and CRVS. Key challenges noted with the implementation of community health ranged across health system dimensions: overall poor leadership and governance, shortage of human resources and other inputs, poor performance of CHWs, and low use of community health services. Many of the responses focused on training CHWs, with some investments in community infrastructure, but overall, very few structural reforms or those related to the software of health systems. Although in some countries significant investments are noted, this remains quite variable, and mostly skewed to CHWs/human resources, with very little left over for broader community engagement and oversight processes. We further contextualise these findings in the sections below.

### Acknowledging and advancing community health

Defining community health can be challenging due to its broad scope, the various approaches that can be taken, and the need for it to be context specific [3]. This may explain why most of countries have not adopted a precise definition in their ICs and PADs. However, although there was no written definition in these documents, the GFF has triggered or supported the development of strategic documents on community health that are driving a common understanding at national level. This helps to establish community health as an essential component of the health system and creates a foundation for its role in improving population health. In fact, community health has often been viewed as distinct from ‘formal health systems’, but there are calls for greater integration to establish ‘community health systems’. This is a promising beginning, but achieving such integration requires further acknowledgement, including explicit definitions, as well as further funding and governance reforms in community health more broadly, in addition to investing in training, supervision, logistical support, and improved working conditions for CHWs [33].

### Addressing CHWs as a foundational element of service delivery

Community health actors, as reflected in these country documents, are diverse, and their names vary from one country to another [34]. The term ‘lay health workers’ or ‘community health workers’ (CHWs) is often used to refer to such actors, and the World Health Organization describes them as ‘health care providers who live in the community they serve and receive lower levels of formal education and training than professional health care workers such as nurses and doctors’ [35]. Formal education, including completion of a particular level, may or may not be required and training to become a CHW may be informal or formal through a recognized institution [36].

Despite significant investments in CHWs cadres (and not to community health more broadly), the ICs and PADs we examined did not provide detailed information regarding their selection criteria, level of training, and remuneration. Selection criteria must consider the nature of the activities that CHWs are required to perform and may include, to varying degree, community membership and embeddedness, reputation and character, level of education, experience with the healthcare system, and gender [36]. Furthermore, gender issues related to CHWs were largely missing from the country documents. Yet, gender issues require special attention because they are often linked to cultural norms, involve social constraints, and can affect the acceptability of services. Additionally, they are sometimes at the root of discrimination against women in terms of remuneration [37–39].

The country documents listed a variety of ways in which CHWs are paid. While some CHWs work as unpaid volunteers, others receive financial compensation or incentives from the state or non-state organizations, sometimes as part of vertical programmes [36], but they are less likely to receive a formal salary like professional health workers. There is a growing consensus that CHWs should receive adequate and formal compensation to enhance their motivation and performance [40–42]. However, in many countries, CHWs continue to be overexploited, underpaid, overworked, under-motivated, and under-supported, which affects the quality of care they provide [40]. CHW incentives are a complex topic and can take many forms with mixed effects on CHW motivation/incentives can be direct or indirect, financial (e.g. salary/stipend including its level and regularity, pension, leave allowance, reimbursement of travel or airtime costs, fellowships, loans) or non-financial (e.g. insurance, role clarity, supportive/facilitative supervision, manageable workload, adequate supplies and working equipment, job security, professional development and career opportunities, formal recognition and trust by their community, the health system and the wider society) [43–45]. To enhance CHWs’ situation and status, context-specific incentives and policies should be developed, accompanied by well-designed reforms, including targeted legislation [41].

As noted in our findings and in the literature, CHWs provide a wide range of activities, whether promotional, preventive, or curative, and can promote equitable access to basic health services [46]. However, these activities vary from country to country, sometimes including specific aspects such as those related to security issues (Burkina Faso, Mali and Niger), climate change (Mali and Niger), or CRVS (Burkina Faso and Guinea). There is evidence that community health can enhance the resilience of health systems in fragile and conflict-affected settings [47,48]. In certain areas of countries with high levels of insecurity, access to and continuity of care pose constant challenges for communities and health systems, and health facilities operate at minimum capacity with very few health staff [49–51]. During times of crisis, CHWs are often the only healthcare providers available [48]. To support their motivation, they require a package of financial and non-financial incentives, including training, supervision, regular input supplies, performance bonuses, and stable remuneration [48,52]. However, implementing these measures in crisis contexts remains challenging.

### Leveraging CHWs embedded knowledge

While often used in instrumental ways by health services, CHWs have broader potential in supporting health promotion and social change in communities. CHWs are usually recruited from within their own community, so they are known and generally accepted by the local population. CHWs’ familiarity with the local environment and people enable them to communicate more effectively, access remote areas and trace population. This asset is valuable for the success of awareness-raising campaigns, including those focused on topics such as climate change [53,54], as planned in the PADs of Mali and Niger. Climate change literacy is relatively low in low- and middle-income countries (LMICs) even though they are the most vulnerable to the adverse health effects of climate change [55–59].

The advantage that CHWs can bring is also likely to have prompted Burkina Faso and Guinea to involve them in CRVS activities, and other countries may consider such an approach to complement their efforts to strengthen CRVS systems. Indeed, despite the importance of CRVS for population surveillance and management, for calculating and monitoring SDGs indicators, including RMNCAH-N, for making evidence-based decisions, and for improving health outcomes [60–64], several gaps still exist in many LMICs [65].

### Community health is not just about CHWs: the need for a holistic approach

Most countries’ ICs and/or PADs identified challenges and weaknesses that can impede the effectiveness of community health interventions. The potential of the proposed interventions to mitigate these challenges and weaknesses and improve community health is undeniable. However, these interventions are similar to what Chee et al. refer to as ‘health system support’ and are limited to the short- to medium-term [66]. To achieve strong and lasting results, genuine health system strengthening is needed, which entails structural reforms and changes [66] and may include the formal integration of community health into the wider health system. Such integration can promote a more holistic approach to community health, rather than one focused on CHWs, as suggested by the funding of community health in the ICs and PADs of the countries studied.

A holistic approach would include supporting: i) other actors (e.g. social workers) providing community-level activities, including health facility outreach activities; ii) community engagement and cooperation to tackle/support social determinants of health, which would need building social partnerships in and outside of the health sector; iii) household production of health, defined as ‘*a dynamic behavioral process through which households combine their (internal) knowledge, resources and behavioral norms and patterns with available (external) technologies, services, information, and skills to restore, maintain and promote the health of their members*’ (p. 206) [67]; iv) systematic integration and consideration of community health in the health information system and in the supply of medicines and medical consumables, or adequate funding for the wide range of community health activities [68]. Community health interventions are effective in terms of reducing morbidity and mortality [69–72]. To ensure their impact, it is important to ensure that funding for community health interventions is not piecemeal or partial.

### Other challenges

With respect to GFF processes in particular, other areas of improvement include the alignment of community health activities between ICs and PADs, as well as the indicators used to monitor their performance and community health activities as a whole, which were not always consistent. This is not ideal and raises questions about the timing and coordination of the ICs and the PADs planning cycles and the steps that need to be taken to ensure greater harmonisation to maximize synergies between them. The PAD, together with other national and external sources of funding that may be identified through a resource mapping process, should ideally fund priorities that have been identified in an inclusive and consensual manner in the IC. The lack of alignment also limits the identification of specific characteristics and/or gaps in the scope and implementation of community health activities that would allow meaningful comparisons and analysis between countries.

### Study limitations and strengths

Although a systematic and rigorous approach was used in this content analysis, the ICs and PADs were the single source of information and this is a limitation of the study because they would not provide a complete picture of the situation. What is written does not necessarily reflect what happens in practice, just as the absence of mention of something does not necessarily mean its absence in practice. Also, the IC and PAD are not expected to have lots of details because they are strategic and not operational level documents. The next step will be to cross-check and supplement with other sources, such as activity or evaluation reports or interviews with key informants involved in the development and/or implementation of these documents. However, our findings remain relevant as they highlight countries’ plans to leverage community health to support RMNCAH-N activities, and this is worth scrutinising to gauge its value and suggest areas for improvement. The authors are health systems researchers who brought their expertise in community health to the analysis and framing of this article. Except for Burkina Faso, they were not involved in developing the country documents assessed. One of the authors serves as an advisory group member to the GFF but remains independent from the GFF, while other authors are familiar with health systems development, financing and planning.

## Conclusions

While francophone West Africa has specific needs, problems and histories, the regional approach to community health has relevance to other LMIC contexts. Analysing inclusion of community health in wider health investments in the region highlights challenges and weaknesses and also raises hopes and offers great potential for improving public health. The GFF has an opportunity to guide on country investments in an effective CHW strategy in the francophone West African countries and catalyse its implementation. A guidance document of how to design and finance an effective CHW strategy could facilitate this goal. Community health plays an important role in the ICs and PADs of the countries studied, underlining its importance as a key approach to improving RMNCAH-N. Nonetheless, community health is often fragmented and poorly integrated into the wider health system. While investments focus on CHWs, much more must be done to strengthen systems to support them and invest more broadly in community health and participation. The GFF has the potential to act as a catalyst in addressing these issues through its inclusive and collaborative approach, mobilizing stakeholders at all levels of the health system and beyond, and streamlining interventions. This must be a much more deliberate priority to effectively mobilize the necessary resources.

## Data Availability

The documents used for this paper are available on the websites of the GFF and the WB.
